# Organisational benefits of undertaking research in healthcare: an approach to uncover impact

**DOI:** 10.1186/s13104-023-06526-5

**Published:** 2023-10-05

**Authors:** Judith Holliday, Natalie Jones, Jo Cooke

**Affiliations:** 1 Research Department, Mid Yorkshire Teaching NHS Trust, Pinderfields Hospital, Aberford Road, Wakefield, WF1 4AL UK; 2 Primary Care Sheffield, Fifth Floor, 722 Prince of Wales Road, Sheffield, S9 4EU UK; 3 School of Health Science, University of Sheffield, 30 Regent Street, Regent Court, Sheffield, S1 4DA UK

**Keywords:** Research impact, Coproduction, Research collaborations, By-products of research

## Abstract

There is increasing focus to review the societal impact of research through assessment and research excellence frameworks. These often link to financial and reputational incentives within the academic community. However, timeframes to demonstrate impact using these approaches are often long and are not designed to show benefit to service collaborators who require evidence of improvement and change to their services more immediately. Impacts that are measured this way may also miss out on unintended and positive impacts that occur as by-products of research, or through the ‘ripple effect’ that research may have on practice. Importantly, demonstrating how research makes a difference can improve the research culture in services, and motivations in service partners to become, and stay involved in research. This article describes, and provides access to, a tool called VICTOR (making Visible the ImpaCT Of Research) that was developed by a community of practice involving 12 NHS organisations through blending evidence from the literature, practice and service users. We describe the types of impact that have been collected by VICTOR and explore how collecting impact in this way might help research-practice partnerships and inform research methodologies and may be useful to show impacts alongside, and shortly after the research process.

**Supplementary Information:**

The online version contains supplementary material available at 10.1186/s13104-023-06526-5.

## Introduction

There is increasing focus within the academic establishment to review the societal impact of research through various assessment and research excellence frameworks. These often link to financial and reputational incentives in academia, for example the research excellence framework in the UK https://www.ref.ac.uk/about/ and Excellence in Research in Australia https://www.arc.gov.au/excellence-research-australia/era-2023). Many governments invest in both applied and basic health research for impact and benefit. The Canadian institute for Health Research (CHIR) for example, aims to develop scientific knowledge into improved health, more effective health services and products, and an effective care system http://www.cihr-irsc.gc.ca/e/37792.html. The UK based National Institute Health Research (NIHR) aims to provide health research that focuses on the needs of patients and the public [1] [2]. However, the timeframes to demonstrate impact from research findings are often very long [3], and many services want to show impact sooner than this resulting in tensions in academic- practice partnerships [4] [5]. There is emerging evidence that there are benefits for healthcare organisations to be part of research delivery in collaborations. For example, hospitals that are research active (defined in terms of linked citations in peer reviewed journals) are associated with improved mortality rates [6], and quality of care and health outcomes positively correlate with the conduct of clinical trials in NHS organisations [7]. There is also an association between research engagement of practitioners and improvements in performance and the process of care [8]. Boaz et al. [9] described these as the ‘by-products’ of research itself, but perhaps it is more than this, and may help to support motivation and engagement of services and increase collaboration with less engaged groups? There is also a growing debate that research could be more immediately beneficial to healthcare providers if conducted in a co-productive manner [10] [11] [12]. Coproduction can stimulate ‘win-win’ and mutually beneficial outcomes in the short-term [13], especially for services and service users and aids longevity of research collaborations and better reach into the healthcare system [14]. Indeed, a realist review focussing on research capacity development in health and care systems has highlighted how showing that research makes a difference can act as an important symbolic mechanism that increases research capacity and research culture in healthcare organisations [15]. Ideally these should be captured contemporaneously within the coproduction process.

## Making visible the impact of conducting research in healthcare organizations: developing the VICTOR tool (making visible the ImpaCT Of research)

With this context in mind, a community of practice (CoP) that included members of Research and Development leaders in 12 NHS organisations in England completed a service development project to develop a tool that would enable the collection of case studies to uncover the immediate impact of conducting research in their organisations. This is more than a ‘by-product’ for them and contributes to quality assessment by the Care Quality Commission and establishes direct benefit to the organisation. The CoP was called ACORN (Addressing Capacity in Organisations Network) and they worked with two NIHR partnerships: The Collaboration and Leadership in Applied Health and Care for Yorkshire and Humber (CLAHRC YH) and the NIHR CRN YH.

VICTOR aimed to identify impact where it matters in the NHS, services, and people within them and to create a resource to support NHS Trusts to capture and show how applied research projects can have an impact within the organisation. Two senior NHS managers (JH and NJ) were seconded into the NIHR partnership to develop the VICTOR approach. Areas of impact were developed through collecting and organising information from a range of sources including a workshop with ACORN members to identify areas they thought were important, that made a difference to services when conducting research. The particular focus was on how undertaking research can make a difference in healthcare organisation and the wider health system.

A scoping literature review was conducted with the aim of understanding the current landscape of research impact tools and mapping out the published tools available for capturing research impact [16]. Keywords were used to systematically search the published literature to identify research, policy, and research impact tools relevant to the project. Online databases such as CINAL and Medline were iteratively searched as well as grey literature. Reports, tools and studies detailing research impact tools were exported to a reference manager so that they could be analysed. NJ and JH then screened the papers to ensure they were relevant to the project. A spreadsheet was created to list the research impact tools and extract data on the key domains of impact. NJ and JH were interested in where the research impact tools were similar, any gaps and the relevance of the tools to the NHS context.

The tools were discussed with JC. The merits of each were analysed. Findings from this review discovered gaps in the patient perspective on research impact and that many of the tools were designed for academic purposes or for contexts other than the NHS. Key tools of interest that were identified were:

- Becker Medical Library Model [17].
- Payback Framework [18].
- Canadian Health Services Policy Research Alliance (CHSPRA) making an impact framework [19.
- Research Excellence Framework [20.
- Sarli CC, Dubinsky EK, Holmes KL. Beyond citation analysis: a model for assessment of research impact [21].

Stakeholder engagement in this project included working with ACORN which included 12 NHS organisations: three teaching hospitals; five mental health trusts; and four acute trusts. Many of these trusts also include outreach into community and public health practice. Each trust has at least two representatives in ACORN, one being a senior R&D manager, and the other a research-active or research interested practitioner. Stakeholder engagement is a powerful tool for involving those in research who have lived insights and ideas about ways to improve healthcare. [22]

Stakeholders in this project were involved in several ways:

i) 12 ACORN NHS trusts met several times during the project to advise on progress and prototype tools.
ii) Experts in the field were consulted about research impact domains via telephone calls.
iii) Patient and carer representatives were consulted about prototype tools one to one and via patient research engagement groups. Feedback was also sought from a mental health charity and an older people’s charity.
iv) Prototyping involved creating versions of the research impact tool and testing them out with stakeholders. Prototyping is a helpful way to test out a new tool in the early stages of development and design. [23]

Feedback on the prototype tools was collated by NJ and JH and used to inform the next version of the tool.

Several patient representatives tested the tool by completing the questions. They used their experiences of participation in a recent study to answer the questions. This gave the authors an understanding of whether the questions were collecting sufficient and focused information. Feedback from patient and informal carer representatives shaped the prototype tool so that the number of questions were reduced to make completing the questionnaire less onerous and the language of the tool was developed to avoid professional jargon.

In the first prototype, the domains of the tool were created by using the data extracted from the scoping review. NJ and JH extracted the key domains from other research impact tools. Information and insights from stakeholder consultation about what needed to be included in the tool were mapped onto the emerging domains. A master domain list was developed and tested out with JC and the ACORN group. Each domain had a list of criteria to define the focus for the domain for example, the ‘health benefits’ domain considers health benefits, safety and quality improvements for research participants and carers. This is that as a result of taking part in the research the participants (patient, carer or family) have improved health, a better experience of care, improved quality of life and/or more equitable access to healthcare. This domain includes the subgroups:

- Health benefits such as; quality of life impacts, access to different treatments; care delivered differently; quality of information provided; health literacy; providing the same quality of care for a reduced cost.
- Experience; during the study, were there any changes made to patient care that improved the experience of care for participants, carers or family as part of / as a result of being in the study for example information giving, carer support, carer interventions; health literacy.)
- Patient safety; are there any examples of improved governance and/or safety for patients taking part in the study? This would include improvements to quality of research in terms of scientific quality, standards of ethics and related management aspects – set up, conduct, reporting and progression towards healthcare improvements.
- Social capital; are participants / carers better connected or part of any new networks as a result of taking part in the research? This includes self-help groups, increased social networks or activities.

By socialising the draft domains we were able to gauge if there were any gaps, duplications, or areas of impact that might have been missed. Feedback shaped version 2 of the list of domains, criteria and prompts which were then used to create questions relevant to the domain criteria. Open questions were developed to elicit information from the research team members or patients [24].

The resulting areas of impact are given in Table 1. There were six general domains of impact, with subgroups within each domain.

**Table 1:** VICTOR: Areas of impact relevant to healthcare organisations

Health Benefit Of Participants	Service & Workforce (made during and/or after project)	Research Profile and Capacity	Economic	Influence	Knowledge Production and Exchange
Health gain of participants during and after projects. Patient experience benefits during project Patient safety gains in during project Equity of access and use of care Social capital (networking of participants) that improves wellbeing	Service changes made Clinical skills developed Workforce changes. E.g. new roles Collective action of service teams or between teams Changes in available products and equipment Changes made in guidelines and clinical processes	Research culture change Research awareness Research Capacity of individuals, teams, services or organisation Networks & collaborations developed and continued Engagement of wider workforce in research (more people delivering and developing research	Cost effectiveness of services Cost savings made Commercial income gained (through undertaking the project itself) Commercialisation (of any outputs from projects- linked to intellectual property)	Cohesion. Do services/ departments work better together? Reputation of organisation (including wider public, other healthcare organisations, academic community) Recruitment and retention of staff Public and patient involvement. How did their involvement make a difference?	Academic Dissemination. Where and how? Knowledge sharing within the organisation and wider afield (may be on methods as well as research findings) New Knowledge identified and used Actionable outputs: outputs from research that are useable: e.g. clinical tools, decision aids, guidelines, training packs.

This framework was then used to develop a questionnaire that was modified and adapted based on two rounds of piloting within the ACORN organisations. A final VICTOR questionnaire was developed that includes 26 questions organised in six sections reflecting the impact domains and domain subgroups described in Table 1. A Tool of four questions was developed for patients and members of the public based on consulting with service user groups. The VICTOR tool can be accessed https://www.e-repository.clahrc-yh.nihr.ac.uk/visible-impact-of-research/ )

As a service evaluation, the project does not require ethical approval through HRA however this project was conducted with the rigour and safeguards of research to protect participants’ data. The service evaluation was registered with the author’s organisation (STH) clinical effectiveness unit. Efforts were made to ensure that this project adhered to best practice guidance for service evaluation practice [25]. Consent to participate in the stakeholder consultations was through explicit verbal or written consent. Those agreeing to view the prototype tool and provide feedback were aware that their feedback data would be used in project reports and dissemination, and all data would be anonymised.

## Uncovering impact: feedback from ACORN trusts through using the tool

Trusts who piloted the VICTOR tool shared their summary documents with the ACORN CoP. Many trusts reported that VICTOR had been helpful in identifying unanticipated and ‘hidden’ impacts of research, and documented changes that would otherwise have been overlooked, or not linked to research activity.

The impacts frequently cited in the pilot sites included service and workforce changes, research capacity building, and health and experiential impacts of patients and carers. Intervention studies often, but not exclusively, produced changes in workforce and services. For example, practitioners who received training as part of developing skills for new interventions frequently highlight how these skills were used in their practice more generally after the research project. These can be diverse skills, like paramedics developing better airway management techniques, or community nurses using cognitive behavioural therapy with patients who have long term conditions. Sometimes elements of the research method were then incorporated into clinical pathways, for example using screening questionnaires in radiography services, or use of autophotography in mental healthcare, where patients use photographs to express their world view or how they feel. The advantages of using such techniques were demonstrated in the research delivery and continued into everyday practice.

Many examples of impact on working practice in the healthcare system were established because of working together on a research project, for example between pharmacy and a clinical area, or between primary and secondary care. These continued to benefit the services after the research had been completed. Such stories were very insightful and meaningful to practitioners and managers, and were able to promote research in the organisation and wider community, for example in newsletters and press releases. Importantly, some patients described impacts that were not mentioned by research teams who were delivering projects, for example patients felt they were closely monitored, felt that they were making a difference, but they also had a contact person, usually the research nurse, who provided support and information about care and services. The process of collecting the information through VICTOR sometimes helped internal cohesion. Informal feedback was collected from the individuals or research teams (collated by NJ and JH) testing out the prototype tools. This suggests that using the VICTOR tool as a team facilitated reflexivity and team thinking about the benefits of the research project, and enabled teams to reflect on the successes of research together. One participant remarked “*Teams don’t usually get together after a research project ends, everyone is getting on with the next project, so it was nice to take some time together and reflect on the project”.*

Another participant comments on the value of the team coming together to collaborate and completing the tool “*We collaborated across a pathway of care, medical, therapy and nursing staff, we would not normally get together to discuss the research, this was helpful as we could discuss changes and improvements in our systems and processes, applying the learning from the study”.*

This strengthened relationships between research and clinical teams by recognising and documenting shared achievements and strengthened the partnerships with researchers. The process also enabled increased awareness of each other’s role and to share their views of impact.

During the prototyping notes of informal feedback suggested that it was more difficult than anticipated for the PI or research coordinator to track down members of the research team to ask them to complete a VICTOR questionnaire. This suggests that doing the feedback directly after the project was concluded could make it easier to gain feedback however this could potentially miss impacts that occur after the study 3–6 months after the project has been completed.

## Outlook and conclusion

The VICTOR tool can help to describe the impact of conducting research in healthcare organisations, and it offers fertile ground for further work and debate on its wider influence. The logic for VICTOR’s development was that by uncovering impact of undertaking research ‘close to practice’, it could show immediate usefulness to clinicians, managers and patients, and stimulate a research culture, triggering a mechanism for change [26]. A report on enabling staff to do research in NHS organisations [27] highlights that feedback on research impact is an enabler to promote a research culture and encourages positive attitudes and values towards research. This may well be more beneficial in in supporting research collaborations within the wider ‘research ecosystem’, particularly in social and community care, where research capacity in needed and where immediate benefits are important and practical benefits realised [28].

There is a growing body of support and funding for long term research and practice collaborations such as the CLAHRCs in England, and the Hunter New England Population health research-practice partnerships [29]. These partnerships provide an opportunity to produce co-benefits to the researchers but currently there is not systematic evidence of how to identify immediate benefits to service partners [30] including methods to capture the intended and unintended outcomes that are context dependant [31]. VICTOR could provide a basis for this. It is argued that impact should be recognised in the eyes of the end- user and be tailored to context of where impact should occur [32] [33] and certainly we have found that hidden benefits have been uncovered through using the tool. The timeframe for VICTOR is undertaken contemporaneously, or shortly after the research and so shows immediate benefit that complements with more longer-term impacts of research collected in the academic research assessment frameworks.

VICTOR also has the potential to determine which research methods and methodologies are valuable to different care provider partners, and help to assess impact and different models of conducting research [30] [29]. Context, for example where coproduction in research is used can influence both process and outcomes [5]. VICTOR has found both stages in research can have a positive and ‘rippled effect’ on service provider organisations further down the pathways to impact and this has also been found by others [34]. Such a body of accumulated knowledge through VICTOR use might help to inform coproduction partnerships providing win-win scenarios linked to process as well as outcomes in research.

We acknowledge that this tool was coproduced with managers, practitioners, and service users in the NHS, which is both a strength and a limitation. It certainly was reported to be useful to the ACORN group and it has been downloaded by hundreds of healthcare organisations. However, it would be beneficial to see if it is useful across the health and care system, or in other countries. There may well be cultural differences in terms of benefit. This calls for more internationally work and comparison and incorporating tools like VICTOR into the research process itself. The optimum timeframe for completing VICTOR was not explored during this evaluation. We hope that by sharing our experience and access to VICTOR we can establish transferability and open dialogue with other partners and provide opportunities to explore mechanisms of impact of research in healthcare organisations.

**Post development note.**

The VICTOR tool and process was made available at https://hseresearch.ie/wp-content/uploads/2021/04/VICTOR-pack.pdf#:~:text=VICTOR%20enables%20engagement%20with%20research%20participants%2C%20professionals%2C%20managers,and%20help%20plan%20for%20improved%20impact%20in%20future. in Feb 2019 and to date 200 organisations have requested a pack. A web based version has been developed and is available at https://sites.google.com/nihr.ac.uk/victor/home and https://victorimpacttool.net/. For further information on accessing the online tool please contact pm.crnyorkshumber@nihr.ac.uk

### Electronic supplementary material

Below is the link to the electronic supplementary material.

Supplementary Material 1

Supplementary Material 2

## Data Availability

Data sharing not applicable to this article as no datasets were generated or analysed during the current study.

## Abbreviations

NIHR: National Institute Health Research
DoH: Department of Health
DH&SC: Department of Health and Social Care
VICTOR: Making Visible the ImpaCT Of Research
CoP: Community of Practice
ACORN: Addressing Capacity in Organisations to do Research Network
CLAHRC YH: Applied Health and Care for Yorkshire and Humber
CRN YH: Clinical Research Network Yorkshire and Humber
