# Supplementary material for: Organisational benefits of undertaking research in healthcare: an approach to uncover impact
Source: BMC Res Notes. 2023 Oct 5;16:255. doi: 10.1186/s13104-023-06526-5 (PMC10557344; doi:10.1186/s13104-023-06526-5)
Supplement: Supplementary file 1 — Supplementary Material 1 [file 13104_2023_6526_MOESM1_ESM.docx]

**VICTOR Impact Questionnaire final**

**Name:**

**Role:**

**Project title:**

**Date of Questionnaire completion:**

**Thank you for agreeing to complete this questionnaire. We are collecting information about the impact of the research study in which you have been involved. We will be combining these answers with those of other people involved across the organisation. This will help to make visible the impact of doing research in our organisation, and to share this with others.**

**We hope to produce impact case studies about the research, and will also produce information for presentations, the annual plan, our website and others.**

**Please read through all the questions before completing the form to help avoid duplication of answers. We are not expecting answers to every question but please ensure the most important aspects of the research impacts are included.**

**As we are speaking to only a handful of people involved in the study, it is possible that what you say will be identifiable so please inform us if you wish for anything to be kept confidential**

Are you happy for your answers to be used in this way? (Please circle a response)

NO

YES

1. **Health benefits, safety and quality improvements for research participants and carers**

As a result of taking part in the research; the participants (patient, carer or family) have improved health, a better experience of care, improved quality of life and/or more equitable access to healthcare.

Question Yes/No/Not Yet Please give examples/describe

| 1. **Health benefit**   Have there been any health benefits to study participants, family or carers as a result of taking part in the study?  (**Prompt**: quality of life impacts, access to different treatments; care delivered differently; quality of information provided; health literacy; providing the same quality of care for a reduced cost. |  |  |
| --- | --- | --- |
| 1. **Experience**   During the study, were there any changes made to patient care that improved the experience of care for participants, carers or family as part of / as a result of being in the study?  (**Prompt**: Information giving, carer support, carer interventions; health literacy.) |  |  |
| **3.Patient safety**  Are there any examples of improved governance and/or safety for patients taking part in the study?  **(Prompt:** Improvements to quality of research in terms of scientific quality, standards of ethics and related management aspects – Set up, conduct, reporting and progression towards healthcare improvements) |  |  |
| 1. **Social capital**   Are participants / carers better connected or part of any new networks as a result of taking part in the research?  (**Prompt**: self-help groups, increased social networks or activities) |  |  |

**B. Service & Workforce impacts**

Since the study has completed, the care that patients in this pathway receive has changed as a result of the research.

As a result of doing the research, the workforce has changed and these changes can be reflected in enhanced clinical skills, increased confidence, quality improvements, changes to job roles and/or changes to job descriptions.

Question Yes/No/Not Yet Please give examples/describe

| 1. **Service change**   Has anyone in the organisation started doing something or stopped doing something clinically as a result of the research?  In addition to this; has this resulted in improved care of patients after the study has finished?  (**Prompt**: quality of life impacts, access to different treatments; care delivered differently; quality of information provided; health literacy – leaflets and guidance) |  |  |
| --- | --- | --- |
| 1. **Clinical or generic skills**   Does anyone have new **clinical skills** as a result of the research? This could include skills developed as a result of being involved in the study or skills that have been developed since the study finished because the benefit of having these skills was demonstrated by the research.  (**Prompt**: any clinical training  Please note research skills are also covered in the next section.) |  |  |
| 1. **Workforce**   Has the workforce changed as a result of the research? For example have there been any changes to job roles or structures?  (**Prompt**: This could be during the study or after the study) |  |  |
| 1. **Collective action**   Has taking part in this research influenced your team to do anything different together?  **(Prompt:** Collective changes to patient care, skills, confidence and/or quality improvements) |  |  |
| 1. **Guidelines**   Is there a different use of, or further adherence to, clinical guidelines as a result of the study – either during the study or afterwards?  (**Prompt**: these could be national guidelines, or those developed more locally as a result of the study.) |  |  |

1. **Research Profile and capacity**

There is organisational, service level and individual research capacity built. Attitudes to research have changed.

Collaborations are built within the organisation and outside the organisation. There is enhanced networking to promote knowledge exchange & build new collaborations.

Questions Yes/No/Not Yet Please give examples/describe below

| 1. **Research culture**   Has the study changed the culture and attitudes to research in the service or organisation?  **(Prompt:** Are you measuring impact now; increased willingness to get involved in research; increase in confidence; Patient and Public Involvement) |  |  |
| --- | --- | --- |
| 1. **Research awareness**   Has staff awareness of research changed as a result of the organisation taking part in this study?  (**Prompt**: Any examples of how this was achieved or is evident?) |  |  |
| 1. **Research capacity**   Has anyone developed new **research skills**, knowledge and experience making them more likely to be involved in future research?  **(Prompt:** New career choices, research roles, individual clinical and research links; collaborations on further grant applications) |  |  |

Questions Yes/No/Not Yet Please give examples/describe below

| 1. **Networks and collaborations**   Has the organisation joined or created any new research networks, partnerships, collaborations as a spin off from the research?  These may be internal or external. |  |  |
| --- | --- | --- |
| 1. **Engagement**   Has the study attracted the interest of others who were not involved?  (**Prompt**: Colleagues in your department, other departments, and/or other organisations?) |  |  |

1. **Economic Impacts**

Has the organisation’s wealth or economic position changed? This would include generating IP, generating income, new tools, spin out companies and cost savings.

Question Yes/No/Not Yet Please give examples/describe below

| 1. **Cost saving / cost effectiveness changes**   Has the adoption of research findings realised any cost savings or promoted cost effective service ( ie same costs better quality of care)? |  |  |
| --- | --- | --- |
| 1. **Commercialisation**   Did the research develop products that generate income or create commercial innovations? |  |  |
| 1. **Income**   Was the study commercially funded and sponsored? Did it generate any income for the organisation?  Did it generate any grant income? |  |  |

**E. Influence**

Has there been an improved reputation of the organisation based on taking part in the research including improved recruitment and retention. There have been changes in the staffs’ attitude, behaviours and awareness of research.

Any evidence of growing leadership in research and teams taking collective action to influence research agendas.

Question Yes/No/Not Yet Please give examples/describe

| 1. **Cohesion**   Has taking part in the research impacted on relationships between professions/ departments/ sectors? |  |  |
| --- | --- | --- |
| 1. **Reputation**   Has taking part in the study had an impact on the profile / reputation of your team or organisation? |  |  |
| 1. **Recruitment and retention of staff**   As a result of the study has there been any impact on recruitment into roles and retention of staff |  |  |

**F: Knowledge Generation and Knowledge exchange**

New knowledge that has been generated by the research is being systematically incorporated into the design, production, adaptation, dissemination and use of information and products to meet user needs.

Ideas are shared and spread and collaborations are built within the organisation and outside the organisation.

Question Yes/No/Not Yet Please give examples/describe

| 1. **Formal dissemination**   Have there been any dissemination events, presentations, conferences or publications about the study, within the organisation or externally? |  |  |
| --- | --- | --- |
| 1. **Knowledge sharing**   Are there any new ways of sharing knowledge within the organisation or between your organisation and others as a result of the research.  **(Prompt:** new groups, networks, face to face/ other media) |  |  |
| 1. **Actionable outputs**   Have any tools useful for practice been developed by the research that the organisation is now using? |  |  |

**G. Anything Else**

If you feel the research study impacted in ways not outlined in the previous questions, please outline your findings here.

|  |
| --- |

Please Outline Your Findings
