# Supplementary material for: Organisational benefits of undertaking research in healthcare: an approach to uncover impact
Source: BMC Res Notes. 2023 Oct 5;16:255. doi: 10.1186/s13104-023-06526-5 (PMC10557344; doi:10.1186/s13104-023-06526-5)
Supplement: Supplementary file 2 — Supplementary Material 2 [file 13104_2023_6526_MOESM2_ESM.docx]

How to use VICTOR

1. **Select a project**.

An individual (usually in the R&D department) identifies a project that has finished or is still underway within the NHS organisation. To start with it is best to try and identify a project that you think is impactful / benefiting your organisation. Consider whether you need to discuss your plans with the Sponsor; studies using NIHR standard agreements require publicity to be agreed by both parties.

1. **Identify a person who will be the Impact Case Study Coordinator (ICC) in your organisation.**

This person will:

- 1. identify people who will complete VICTOR
  2. plan the collection of information via the questionnaire, and summarise the information provided by the completed questionnaires
  3. make sure that the findings are shared with the right people, and the information uncovered by VICTOR goes to the right places.
  4. the ICC may also help with further impact planning with the clinical and research teams

1. **The Impact ICC identifies people who can complete elements of the VICTOR case study.**

This can include any of the groups identified in the table below.

Make sure if you collect information from patient/ carer research participants, you use an approach that is in accordance with governance procedures of the Trust and project ethical approval. It may only be possible to collect information with user and patient research participants for projects that are on-going during their regular contacts with the Trust. However, our experience is that it really is worthwhile to give participants a chance to share their experiences and provide impactful stories.

| **VICTOR Contacts** | | | |
| --- | --- | --- | --- |
| **Principal Investigator:** The person in charge of running the study in the Trust. | |  | |
| **A research team member**; A nurse, Allied Health Professional, member of the medical team or ward staff. These people recruit patients and introduce the study to the patient. |  | **Patient, Family and Carers** |  |
| **Research Manager:** This person has an overview of the studies in the Trust, links to senior management in the Trust and links to wider research networks external to the Trust. |  | **Industry Partner: Not all studies have an industrial partner. However, if** there is an industrial partner; this might include firms linked to technology development, data linkage and drug development. |  |

1. **The ICC invites the VICTOR contact to complete the relevant elements of the VCTOR tool**. The ICC can either:
2. interview the contact and use VICTOR as a structured interview schedule and record the answers on the word document or paper copy of the questionnaire
3. asks the VICTOR contact to complete the word document in their own time and send back to them

The ICC is reminded to:

- 1. please ask the VICTOR contact to provide as much detail as possible when giving YES responses to any of the questions. The prompts may help with shaping these detailed descriptions based on the contacts experience in the project. Evidence like tweets, pictures/ films and photos as well as a description can be useful provide evidence of impact
  2. for any ‘NOT YET’ responses, the ICC should discuss with the VICTOR contact to plan further impact and log this. This can then be used to see the impact planning form.

1. **Patient, family and carer engagement**.

Research participants have their own questionnaire which was created with service user input. How you identify and plan to engage with research participants should be undertaken in accordance with your governance processes and follow up plans in the study. Some ways that people have engaged research participants includes as part of regular follow up visits, Patient and Public Involvement steering group members who have agreed to be contacted in the future during completion of Patient Research Experience Surveys (PRES).

1. **Engaging with Industry.**

If the study has an industry partner the main questionnaire can be used in the same way as with colleagues within the Trust.

1. **The ICC collates the questionnaire responses**.

These are then summarised into the summary template. The summary template should be shared with the VICTOR contacts to ensure that they are happy with this summary before this is used more widely.

|  |  |
| --- | --- |

1. **Use the findings to highlight the impact of doing research in your organisation**.

This might include in Board presentations, evidence for QCQ submissions (research is not within the ‘well led’ framework), share with communications teams for internal and external sharing via social media promotion, press releases, websites etc. You could also discuss with study teams internally and externally to inform future research. Some of this evidence might be useful for researchers in that it can provide testimonials of benefit in the ‘real world’. This may also be helpful for further research planning.

1. **Action planning about further impact**.

Where the ‘NOT YET’ responses have been given by the VICTOR contact, the ICC can work with them to plan and maximise contact using the Impact Planning Template.

1. **Further collection.**

Some teams plan to use the VICTOR tool more than once with the same project to uncover benefits of doing research over time during and after the project has finished.

This documentation has been produced as part of the VICTOR study funded and supported by NIHR CLAHRC Yorkshire and Humber, hosted by Sheffield Teaching Hospitals NHS FT © 2018
